# Expression of Caytaxin Protein in Cayman Ataxia Mouse Models Correlates with Phenotype Severity

**DOI:** 10.1371/journal.pone.0050570

**Published:** 2012-11-30

**Authors:** Kristine M. Sikora, LaGina M. Nosavanh, Prameela Kantheti, Margit Burmeister, Michael Hortsch

**Affiliations:** 1 Program in Cellular and Molecular Biology, University of Michigan, Ann Arbor, Michigan, United States of America; 2 Molecular & Behavioral Neuroscience Institute, University of Michigan, Ann Arbor, Michigan, United States of America; 3 Department of Psychiatry and Human Genetics, University of Michigan, Ann Arbor, Michigan, United States of America; 4 Department of Cell and Developmental Biology, University of Michigan, Ann Arbor, Michigan, United States of America; University of Edinburgh, United Kingdom

## Abstract

Caytaxin is a highly-conserved protein, which is encoded by the *Atcay/ATCAY* gene. Mutations in *Atcay*/*ATCAY* have been identified as causative of cerebellar disorders such as the rare hereditary disease Cayman ataxia in humans, generalized dystonia in the dystonic (dt) rat, and marked motor defects in three ataxic mouse lines. While several lines of evidence suggest that Caytaxin plays a critical role in maintaining nervous system processes, the physiological function of Caytaxin has not been fully characterized. In the study presented here, we generated novel specific monoclonal antibodies against full-length Caytaxin to examine endogenous Caytaxin expression in wild type and *Atcay* mutant mouse lines. Caytaxin protein is absent from brain tissues in the two severely ataxic *Atcay^jit^* (*jittery*) and *Atcay^swd^* (*sidewinder*) mutant lines, and markedly decreased in the mildly ataxic/dystonic *Atcay^ji-hes^* (*hesitant*) line, indicating a correlation between Caytaxin expression and disease severity. As the expression of wild type human Caytaxin in mutant *sidewinder* and *jittery* mice rescues the ataxic phenotype, Caytaxin’s physiological function appears to be conserved between the human and mouse orthologs. Across multiple species and in several neuronal cell lines Caytaxin is expressed as several protein isoforms, the two largest of which are caused by the usage of conserved methionine translation start sites. The work described in this manuscript presents an initial characterization of the Caytaxin protein and its expression in wild type and several mutant mouse models. Utilizing these animal models of human Cayman Ataxia will now allow an in-depth analysis to elucidate Caytaxin’s role in maintaining normal neuronal function.

## Introduction

Caytaxin is a neuron-restricted protein encoded by the gene *Atcay/ATCAY.* Mutations in the human *ATCAY* gene cause the rare neurological disorder Cayman ataxia [1], which is a congenital, non-progressive form of cerebellar ataxia characterized by marked motor defects and mental retardation [2]. While Caytaxin expression has not been characterized in individuals with Cayman ataxia, mutations in *Atcay*/*ATCAY* are predicted to decrease or eliminate Caytaxin expression within the nervous system. Despite this evidence, which suggests a critical role for Caytaxin in normal brain activity and motor control, the function of the Caytaxin gene protein product remain unknown.

Three ataxic mouse mutants have been identified that harbor unique mutations in the mouse homologue of *ATCAY* (*Atcay)* [1]. These mutations all arose spontaneously in different regions of the gene, and resulted in similar but distinct phenotypes. *Atcay^ji^* (*jittery*) is caused by a B1 element (retrotransposon) insertion in exon 4 of the *Atcay* gene, which is predicted to result in a reduction of mRNA levels and truncation of Caytaxin [1]. Mice homozygous for the mutation display severe trunk and limb ataxia which hinders their ability to mate and access food and water, often resulting in starvation and dehydration soon after weaning [1], [3]. *Atcay^swd^* (*sidewinder*) mutants are phenotypically similar to *jittery* but harbor a 2 base pair deletion in exon 5 of *Atcay,* which, is expected to produce a truncated non-functional protein due to a frame shift [1]. This also leads to a drastic reduction in mRNA, likely due to nonsense-mediated decay [1]. *Atcay^ji-hes^* (*hesitant*) mutant mice differ from *jittery* and *sidewinder* in that they display a mild form of ataxia accompanied by dystonia. These mice have a normal life span and are able to reproduce and rear offspring [4]. *hesitant* mutant mice are hypomorphs due to an intracisternal A particle (IAP) insertion in intron 1 of *Atcay*, which affects splicing. Similarly, the mutant *dystonic* rat strain (SD-dt:JFL) also harbors an IAP insertion in intron 1 of the *Atcay* rat homologue, resulting in a marked reduction of cerebellar Caytaxin levels [5], [6]. While these known rodent and human *Atcay/ATCAY* mutations differ in gene location and how they affect the genomic sequence and its protein product, each results in pronounced defects in motor coordination. Although all mouse *Atcay* mutations are predicted to decrease or eliminate Caytaxin protein expression [1], the effects of different mutations on Caytaxin expression has not been characterized.

While the role of Caytaxin in the nervous system remains poorly understood, protein homology mapping has identified highly conserved domains that may be critical for its function. The most notable of which is the BNIP-2/Cdc42GAP homology (BCH) domain, which is involved in a wide range of cellular functions through its interaction with small GTPases, such as GAPs and GEFs [7], [8], [9]. Phylogenetic analysis of various vertebrate Caytaxin orthologs indicates a high level of amino acid conservation within the BCH domain [10], which suggests a potentially shared physiological function across species. Several studies have characterized Caytaxin protein-protein interactions, which depend on its conserved domains and which may provide clues into its physiological role within the nervous system [11], [12], [13], [14], [15].

Buschdorf *et al.* have identified both peptidyl-prolyl cis/trans-isomerase (Pin1) and kidney-type glutaminase (KGA) as proteins that directly bind Caytaxin [12], [13]. Pin1, a factor that is known to affect structure, phosphorylation status, and stability of proteins involved in cell cycle control, interacts with the Caytaxin protein during neuronal differentiation through MEK2 (MAPK kinase) [12]. Additionally, Aoyama *et al.* have shown that kinesin light chain (KLC) directly binds and transports Caytaxin along axons towards distal regions of neurons by a microtubule- and kinesin-dependent mechanism [11]. Investigations into the localization of Caytaxin protein have confirmed preliminary results by Bomar *et al.* that predicted Caytaxin expression predominantly in the nervous system [1], [13], [16], and also found Caytaxin protein concentrated at presynaptic sites of GABAergic neurons [13], [16].

While these data suggest that Caytaxin plays an important role at synapses and/or during neuronal differentiation, it remains unclear how mutations in the *Atcay* gene affect the ability of Caytaxin to properly function and maintain normal motor control. To date, there has been no published characterization of Caytaxin expression in mutant *Atcay* mouse models that provide insight into the function of Caytaxin *in vivo*.

In advancing toward an understanding of how Caytaxin normally functions within the nervous system, we sought to characterize Caytaxin expression in the *sidewinder, jittery,* and *hesitant* mouse lines using novel monoclonal antibodies (mAbs) specific for Caytaxin. We also investigated the potentially conserved function of Caytaxin by rescuing the ataxic phenotype in transgenic *sidewinder* and *jittery* mice through over-expression of human *ATCAY*. Through the use of mouse models, which display an assayable phenotype resulting from mutations in *Atcay*, we demonstrate that a more link between Caytaxin expression and function can be established. Further investigation into motor defects in these mouse models represents a promising approach in elucidating the physiological function of Caytaxin within the nervous system.

## Results

### Monoclonal Antibodies Generated Against Full-length Caytaxin Detect Multiple Protein Bands

To examine Caytaxin protein expression, mAbs were generated against full-length human Caytaxin protein (described in Materials and Methods). A human Caytaxin fusion protein was expressed and purified from bacteria, which was subsequently used as an immunogen in mice to ultimately generate anti-Caytaxin secreting hybridoma cell lines. Using SDS polyacrylamide gel electrophoresis (SDS-PAGE) and a Western blot-based screen, whole brain lysate from wild type C57BL/6J mice were probed with hybridoma cell line supernatants (Fig. S1), and compared to polyclonal antiserum that was obtained from the immunized mouse used for the generation of hybridoma cell lines (Fig. S1, lane 2). This polyclonal antiserum reacts with three distinct protein bands ranging in size from 50–60 kDa and an additional protein band of approximately 67 kDa. Hybridoma cell line supernatants 1E2, 4E3, and 8F4 all react with the same three 50–60 kDa protein bands, which are also detected by the polyclonal anti-Caytaxin antiserum, but did not react with the 67 kDa band (Fig. S1, lanes 3–5). As none of the 3 independent mAbs recognizes this 67 kDa protein and as no transcripts potentially encoding a 67 kDa Caytaxin isoform have been detected or described, we assume that it represents an antigen that is recognized by the polyclonal antiserum and is not related to the Caytaxin protein.

Based on their amino acid sequences, the calculated molecular weights for human and mouse Caytaxin are 42.12 kDa and 42.18 kDa, respectively [17]. The three protein bands, which are detected by the polyclonal antibody and the three selected hybridoma supernatants, have apparent molecular weights that are not only larger than 42.18 kDa, but appear as three distinct bands. The two larger protein species are 58 kDa and 55 kDa in apparent molecular size and the smallest protein band is around 50 kDa. Since all three of these bands were not recognized by control hybridoma supernatants (Fig. S1, lanes 6 & 7), we hypothesized that the three selected hybridoma supernatants are specific for Caytaxin and detect three different Caytaxin isoforms. Thus, hybridoma cell lines 1E2, 4E3 and 8F4 were sub-cloned and further investigated as potential candidates for secreting antibodies specific for the Caytaxin protein.

### Caytaxin Protein Expression is Absent or Reduced in Homozygous Atcay Mouse Mutants

We next sought to determine the specificity of our selected mAbs and to confirm that all three protein bands, detected by the hybridoma supernatants, represent Caytaxin protein. The mutant *Atcay* mouse lines, *sidewinder*, *jittery*, and *hesitant* each harbor unique mutations in the mouse *Atcay* gene, which were predicted to affect Caytaxin protein expression [1]. Homozygous *jittery* and *sidewinder* mutant mice display a severely ataxic phenotype (Movie S1) and were predicted to produce a truncated non-functional Caytaxin protein [1]. In contrast, homozygous *hesitant* mutant mice exhibit a mild ataxic/dystonic phenotype (Movie S2) and due to a defect in RNA processing, were expected to have a reduced level of Caytaxin protein [1].

**Figure 1:**
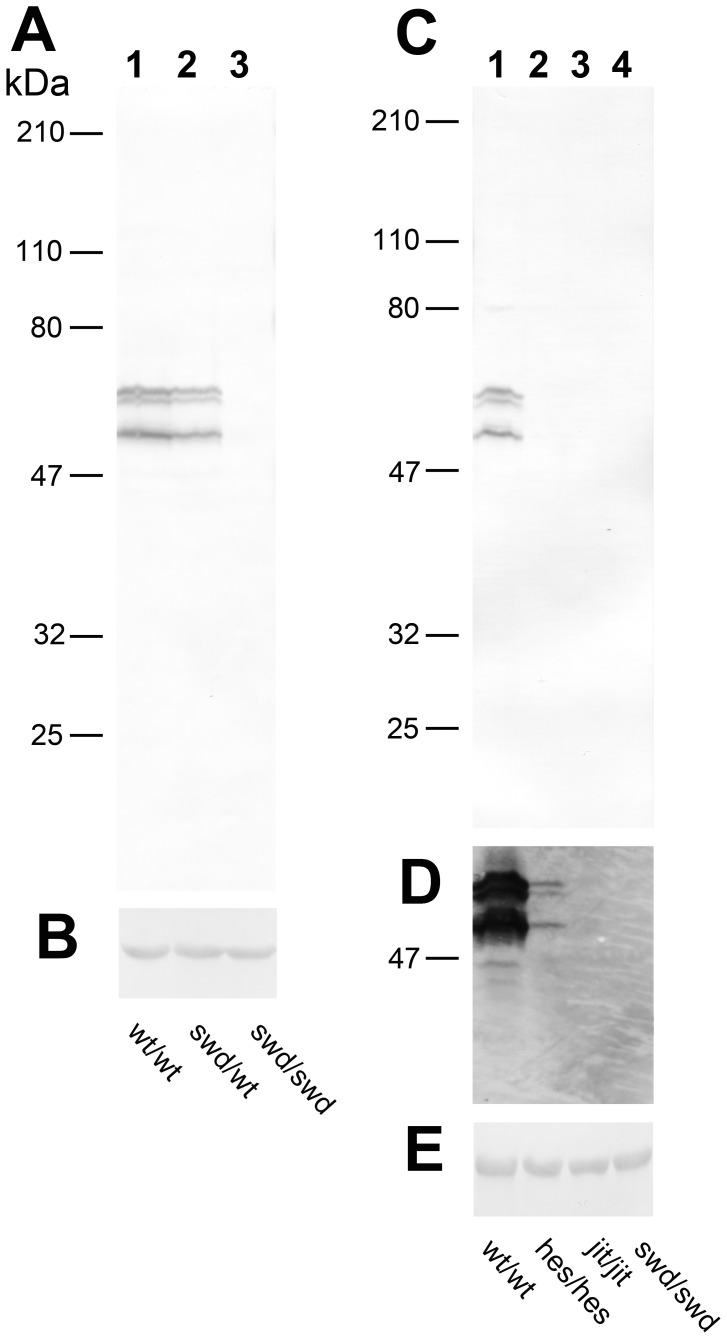
Caytaxin protein expression in *Atcay* mouse mutants. (**A**) Western blot depicting Caytaxin expression in *sidewinder* littermates using anti-Caytaxin mAb 8F4, developed with DAB. Lane 1, wild type (wt/wt); lane 2, heterozygote (*swd*/wt); lane 3, homozygote (*swd*/*swd*). (**B**) Represents a segment of the blot shown in (A) that was probed with an antibody specific for beta-actin. **(C)** Caytaxin expression in *Atcay* mutant mouse lines using anti-Caytaxin mAb 1E2, developed with DAB. Lane 1, wildtype (wt/wt); lane 2, *hesitant* mutatnt (*hes*/*hes*); lane 3, *jittery* mutant (*ji/ji*); lane 4, *sidewinder* mutant (*swd*/*swd*). (**D**) Blot from (B) re-probed with mAb 1E2 and developed with ECL. (**E**) Displays a segment of the immunoblot shown in (C) and (D) that was probed with an antibody against beta-actin. All lanes contain 60 µg total protein from frozen mouse brain lysates.

A comparison of the Caytaxin protein expression pattern of wild type versus affected littermates from these mutant lines strongly suggests that all three protein bands detected by our mAbs are indeed Caytaxin (Fig. 1). The Western blot shown in Fig. 1A contains total brain lysates from wild type (wt/wt), heterozygous (*swd*/wt), and mutant (*swd*/*swd*) littermates from the *sidewinder* mouse line respectively. In wild type mice, all three putative Caytaxin protein isoforms are strongly expressed, but are absent in severely ataxic homozygous *swd*/*swd* littermates (Fig. 1A, lanes 1 & 3). Caytaxin protein expression was decreased in heterozygous littermates, which harbor one normal allele but do not show signs of ataxia nor dystonia (Fig. 1A, lane 2). This result demonstrates that each of the three bands detected by our mAbs is Caytaxin protein.

Similar to *swd*/*swd* mice, mice homozygous for the *jittery* mutation (*ji*/*ji*) do not express Caytaxin protein (Fig. 1C & D, lanes 3 & 4). In contrast, *hesitant* mutant mice (*hes*/*hes*) express Caytaxin protein at a very low, but detectable level – more than 10-fold reduced, when compared to wild type littermates (Fig. 1D, lanes 1 & 2). Caytaxin protein was only detectable in protein extracts from *hesitant* homozygous mice after over-exposing ECL-probed Western blots (Fig. 1D, lane 2).

### Multiple Caytaxin Isoforms are Observed Across Species

**Figure 2:**
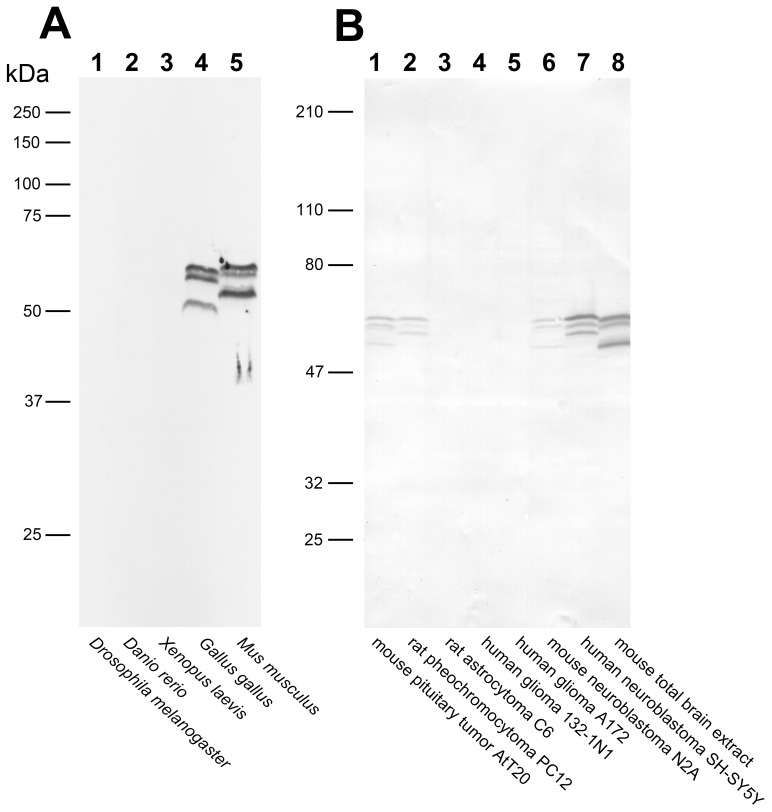
Caytaxin protein expression in various species and cell lines. Each lane contains 60 µg total protein. (**A**) Lane 1, total *Drosophila* embryo extract; lane 2, Zebrafish nervous system; lane 3, *Xenopus laevis* adult brain; lane 4, chicken brain; and lane 5, mouse adult brain. Probed with anti-Caytaxin mAb 1E2 and developed with ECL. (**B**) Total protein lysates from frozen cell line pellets. Lane 1, AtT20 (mouse corticotroph pituitary tumor); lane 2, PC12 (rat pheochromocytoma); lane 3, C6 (rat astrocytoma); lane 4, 132-1N1 (human glioma); lane 5, A172 (human glioma); lane 6, N2A (mouse neuroblastoma); lane 7, SH-SY5Y (human neuroblastoma); and lane 8, wild type mouse brain. Probed with anti-Caytaxin mAb 8F4 and developed with DAB.

To characterize the trans-species reactivity of our anti-Caytaxin mAbs and probe for the existence of multiple Caytaxin protein isoforms in other animal model systems, Caytaxin protein expression was examined by Western blotting in cell lysates from *Drosophila melanogaster*, *Danio rerio*, *Xenopus laevis*, *Gallus gallus*, and *Mus musculus* (Fig. 2A). This analysis revealed no cross reactivity of our antibodies with the Caytaxin orthologs in the fruit fly, zebrafish, or frog (Fig. 2A, lanes 1–3); likely due to a lower amino acid sequence similarity with human Caytaxin (50%, 77%, and 86%, respectively). However, our mAb detected Caytaxin in chicken nervous system protein extracts (an 88% amino acid sequence similarity with human Caytaxin) as three separate protein isoforms (Fig. 2A, lane 4).

We next assessed whether Caytaxin protein expression is restricted to specific cell types within the nervous system and if our mAbs are able to detect human Caytaxin protein. Protein extracts from a panel of neural cell lines were analyzed using Western blots. Caytaxin protein was detected in mouse pituitary tumor and in rat adrenal medulla pheochromocytoma cells (Fig. 2, lanes 1 & 2), as well as human and mouse neuroblastomas (Fig. 2, lanes 6 & 7). However, we were unable to detect any Caytaxin protein in non-neuronal rat cells or human glioma cell lines (Fig. 2B, lanes 3–5).

Our analyses performed with both mAbs 1E2 and 8F4 consistently detected a pattern of three differently-sized Caytaxin protein isoforms. These three isoforms were observed in nervous system and neuronal cell samples from bird and various mammalian species, including humans. The apparent molecular weight of the two largest Caytaxin isoforms is very similar in all species tested. However, the apparent molecular weight of the third and smallest isoform is higher in human and rat samples (Fig. 2B, lanes 2 & 7) when compared to chicken and mouse tissues (Fig. 2A, lanes 4 & 5).

### The Two Largest Caytaxin Isoforms Originate from Alternative Methionine Start Sites

Western blot analyses consistently detect Caytaxin as three distinct protein isoforms in unique, species-specific patterns. Therefore, we tested whether these three Caytaxin protein isoforms are the result of either RNA splicing or co- or post-translational protein modifications. Potential protein modifications were predicted based on the amino acid sequences of both mouse and human Caytaxin using online tools to search sites vulnerable for sumoylation (http://www.abgent.com/tools/), glycosylation, sulfation, phosphorylation, acetylation, and ubiquitination [18], [19], [20], [21], [22]. Though these tools predicted the possibility of phosphorylation and ubiquitination, Western blot analyses using anti-Ubiquitin antibodies and experiments employing lambda-phosphatase excluded both as causes of the multiple Caytaxin protein products (data not shown).

**Figure 3:**
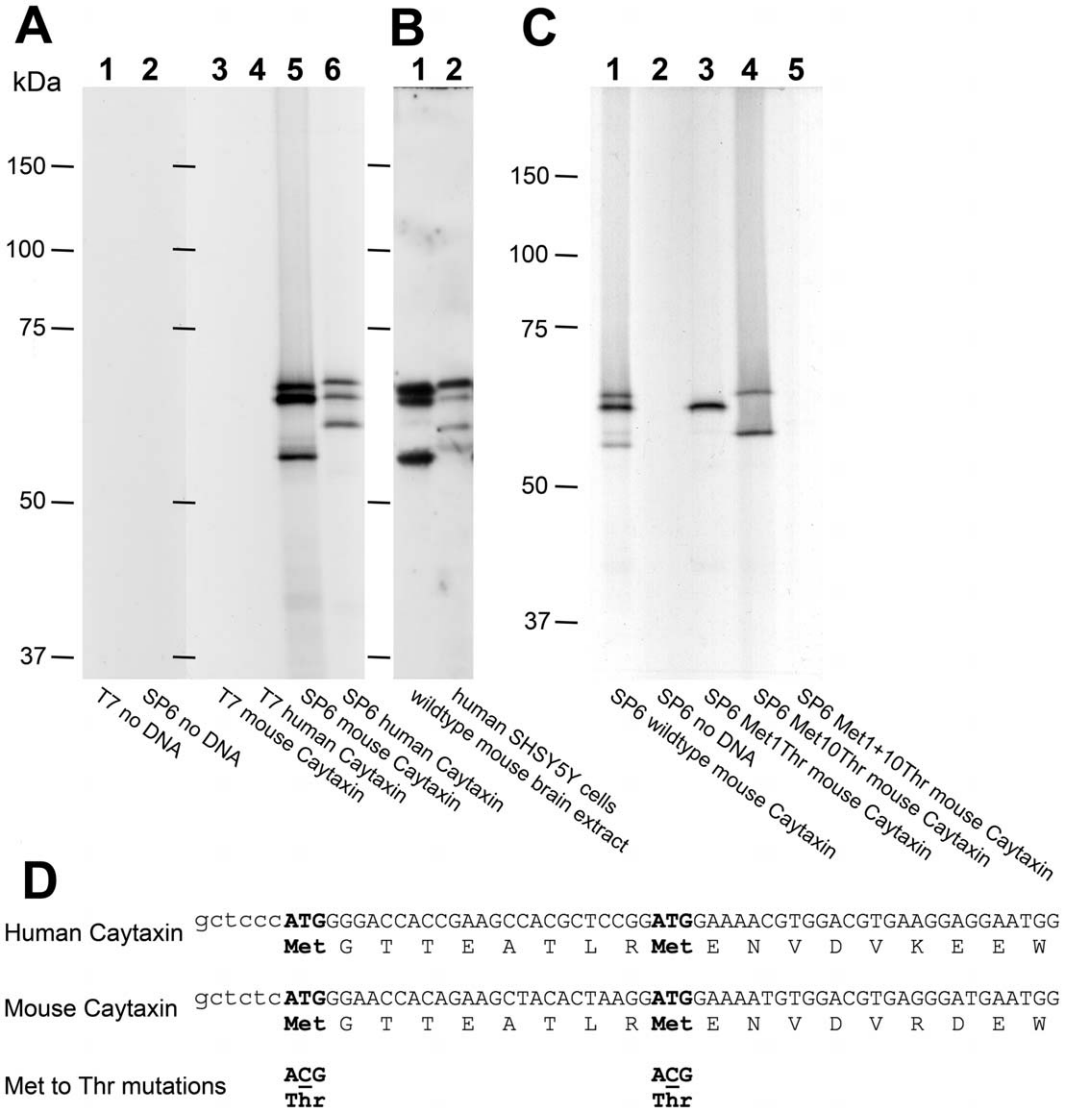
In vitro translation of wild type and mutant *Atcay/ATCAY* cDNAs. (**A**) shows an autoradiogram of Caytaxin protein that was produced from cDNA by *in vitro* transcription/translation as described in Materials and Methods. Lane 1, T7 RNA Polymerase, no DNA; lane 2, SP6 RNA polymerase, no DNA; lane 3, T7 RNA polymerase, mouse *Atcay* cDNA; lane 4, T7 RNA polymerase, human *ATCAY* cDNA; lane 5, SP6 RNA polymerase, mouse *Atcay* cDNA; and lane 6, SP6 RNA polymerase, human *ATCAY* cDNA. (**B**) Western blot from an adjacent part of the gel depicted in (A), incubated with anti-Caytaxin mAb 8F4 and stained with ECL. Lane 1, 30 µg wild type mouse brain and lane 2, 1×10^6^ human neuroblastoma SHSY5Y cells. (**C**) Caytaxin protein from methionine mutant cDNA constructs by *in vitro* transcription/translation using SP6 RNA polymerase. Lane 1, wild type mouse *Atcay* cDNA; land 2, no DNA; lane 3, mutation of first methionine; lane 4, mutation of second methionine; and lane 5, mutation of both methionine residues. (**D**) Nucleotide and protein amino acid sequence comparison between mouse and human Caytaxin. The two conserved methionine residues (Met1 and Met 10), which are separated by 8 amino acids, are marked in bold.

To elucidate the origin of the multiple Caytaxin protein isoforms, we employed a mammalian *in vitro* transcription/translation protein expression system. Using this system eliminates most post-translational protein modifications or differential RNA splicing processes, thus identifying only native unmodified Caytaxin protein. *In vitro* transcripts were generated from plasmid constructs containing the entire mouse or human *Atcay/ATCAY* open reading frame under control of an SP6 promoter. Subsequently, *in vitro*-translated *Atcay/ATCAY* mRNAs were translated in the presence of ^35^S-labeled methionine using a rabbit reticulocyte lysate. Radioactively labeled protein products were separated by SDS-PAGE and detected by autoradiography (Fig. 3A). Separate reactions without DNA or with T7 RNA polymerase were used as negative controls and did not result in detectable protein products (Fig. 3A, lanes 1–4). Both mouse and human *Atcay/ATCAY* cDNA constructs produce the identical three-protein-isoform patterns *in vitro* (Fig. 3A, lanes 5 & 6) that are observed on Western blots of wild type mouse brain and human SH-SY5Y neuroblastoma cell protein lysates (Fig. 3B).

These results, combined with the high similarity of the protein sequences in various Caytaxin orthologs, suggest that the origin of the protein isoforms resides in the DNA/mRNA or protein sequence. An amino acid alignment of human and mouse Caytaxin protein sequences revealed two highly conserved methionine residues (Met1 and Met10) at the predicted amino terminus separated by 8 amino acids (approximately 5.5 kDa) (Fig. 3D). Both ATG codons adhere to Kozak consensus sequence predictions and could account for two separate protein translation start sites. Given the consistency of expression and the conserved apparent molecular weight of the two largest protein isoforms, we sought to determine whether these methionine residues could both serve as protein translation start sites.

Using site-directed mutagenesis, we created single and double nucleotide changes in the Met1 and Met10 methionine-encoding ATG codons in mouse *Atcay* cDNA by converting them to ACG codons (threonine) (Fig. 3D). Each mutation was designed to abolish the ability of the respective methionine residue(s) to serve as (a) translation start site(s), thereby resulting in the absence of the corresponding protein band(s). Three mutant *Atcay* cDNA constructs (Met1Thr, Met10Thr, and the double mutant Met1+10Thr) were tested in the coupled transcription/translation system (Fig. 3C), with wild type mouse *Atcay* cDNA serving as a positive control. Caytaxin protein translated from the Met1Thr construct (mutation of the first methionine Met1) abolished the largest Caytaxin band, as expected, however only the second largest protein band remained visible (Fig. 3C, lane 3). Translation from the Met10Thr construct (mutation of the second methionine Met10) produced a Caytaxin product lacking only the second largest Caytaxin band. However, the Met10Thr mutation was also accompanied by an upshift of the size of the third protein species compared to the corresponding band in wild type *Atcay* cDNA construct (Fig. 3C, lane 4 compared to lane 1). Finally, consistent with results from the single-mutant constructs, the construct harboring both mutations Met1+10Thr failed to produce any detectable Caytaxin protein (Fig. 3C, lane 5).

Common non-methionine start sites, leucine (Leu) and valine (Val), downstream of the second conserved methionine were identified as potential alternative start codons [23], and were also examined (Leu24, Val34, Leu37, and Val41). Similar to methods used to test Met1 and Met10, point mutations were created to disrupt each potential non-methionine start site and the cDNA was processed *in vitro* to observe the resultant translated protein. While the point mutations did have some effect on the apparent molecular weight and/or intensity of this third Caytaxin protein band, none of the sites tested could be confirmed as a starting amino acid of the third variable isoform (data not shown). Additionally, employing a similar method to cleave 58 residues from the C-terminus of Caytaxin, excluded whether the third isoform was produced from C-terminal protease cleavage of either of the larger isoforms as no effect on the third Caytaxin protein band was observed (data not shown).

### Caytaxin Expression in the Mouse Nervous System Peaks at 3 Months After Birth

We found no significant differences in the ratio of each isoform relative to the others upon examination of Caytaxin protein expression in major brain regions from wild type mice (Fig.S2) or among different neuronal cell types. Therefore, we analyzed Caytaxin levels from postnatal day 1 throughout adulthood to examine whether the expression of the three isoforms is subject to regulation throughout postnatal development.

**Figure 4:**
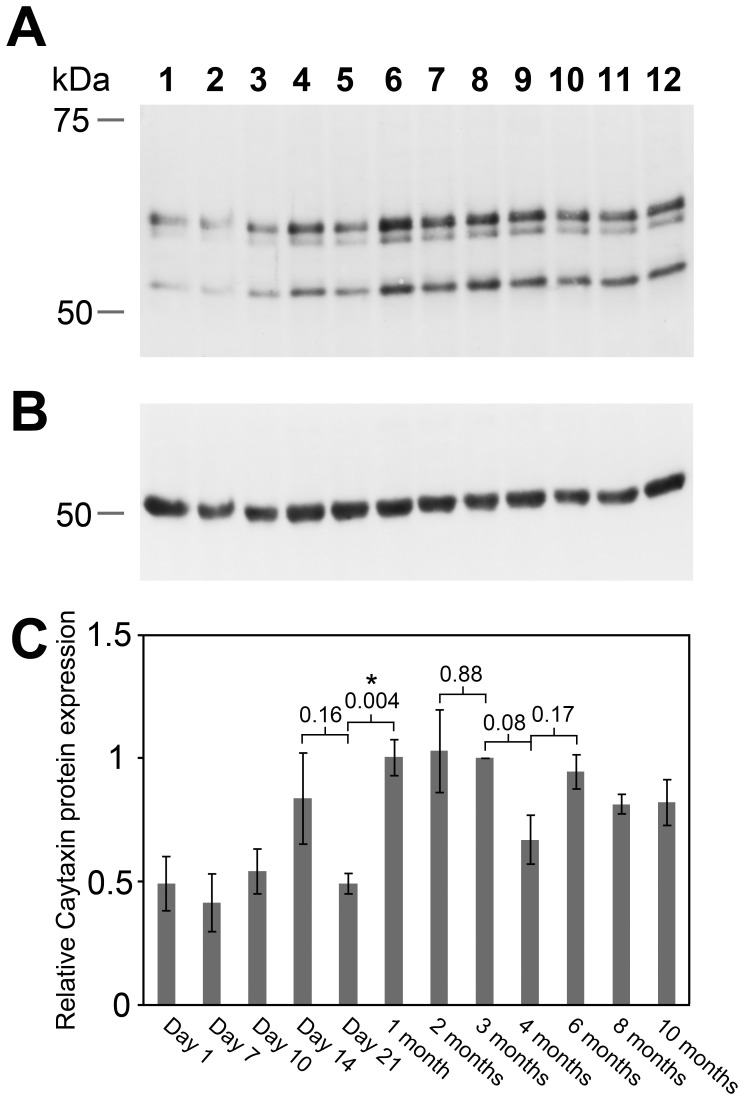
Caytaxin protein expression throughout mouse brain development. Each lane contains 30 µg of total brain protein extract obtained from wt/wt *sidewinder* mice at post-natal ages Day 1 (lane 1), Day 7 (lane 2), Day 10 (lane 3), Day 14 (lane 4), Day 21 (lane 5), 1 month (lane 6), 2 months (lane 7), 3 months (lane 8), 4 months (lane 9), 6 months (lane 10), 8 months (lane 11), and 10 months (lane 12). (**A**) Western blot probed with anti-Caytaxin mAb 8F4 and re-probed with anti-β-tubulin as a loading control (**B**). **(C)** Protein band density calculations described in materials and methods. Densities were averaged among all mice per time point (n = 3, except at 10 months where n = 2). Caytaxin was normalized to β-tubulin and plotted relative to average density at 3 months. P-values generated using Student’s t-test. Error bars generated based on standard distribution from the mean of each group.

Protein lysates were prepared from whole brains of wild type mice at various ages ranging from post-natal day 1 to 10 months, and analyzed by Western blots. Caytaxin protein levels were determined relative to tubulin protein expression from the same sample (Fig. 4, A & B). All Caytaxin protein isoforms were consistently expressed at comparable ratios at each time point (Fig. 4A). Although amount of total Caytaxin varied somewhat between individual mice for most time points, a steady temporal pattern was maintained overall. Similar to previous reports [5], [16], total Caytaxin protein levels were modulated during postnatal rodent development (Fig. 4C). Shortly after birth, Caytaxin protein is expressed at its lowest postnatal level (Fig. 4, lanes 1 & 2) and begins to rise around postnatal day 10 to its highest level around 1–3 months (Fig. 4, lanes 3 & 6–8). At this point, protein expression is largely stable at a robust level until it decreases slightly at the more advanced ages of 10 months and beyond (Fig. 4, lane 12). No significant differences in the relative quantities of the three Caytaxin isoforms were observed during postnatal development.

### The Function of Mouse and Human Caytaxin is Conserved

The Caytaxin family of proteins shares a high degree of amino acid sequence conservation, including a well-preserved BCH domain, which appears to play important roles in protein-protein interactions and function [7], [8], [9]. An amino acid alignment of the mouse and human Caytaxin protein sequences reveals a 91% overall sequence similarity and a 96% sequence similarity between BCH domains (not shown). This high degree of sequence homology suggests that the physiological function of mammalian Caytaxin proteins may also be conserved.

**Figure 5:**
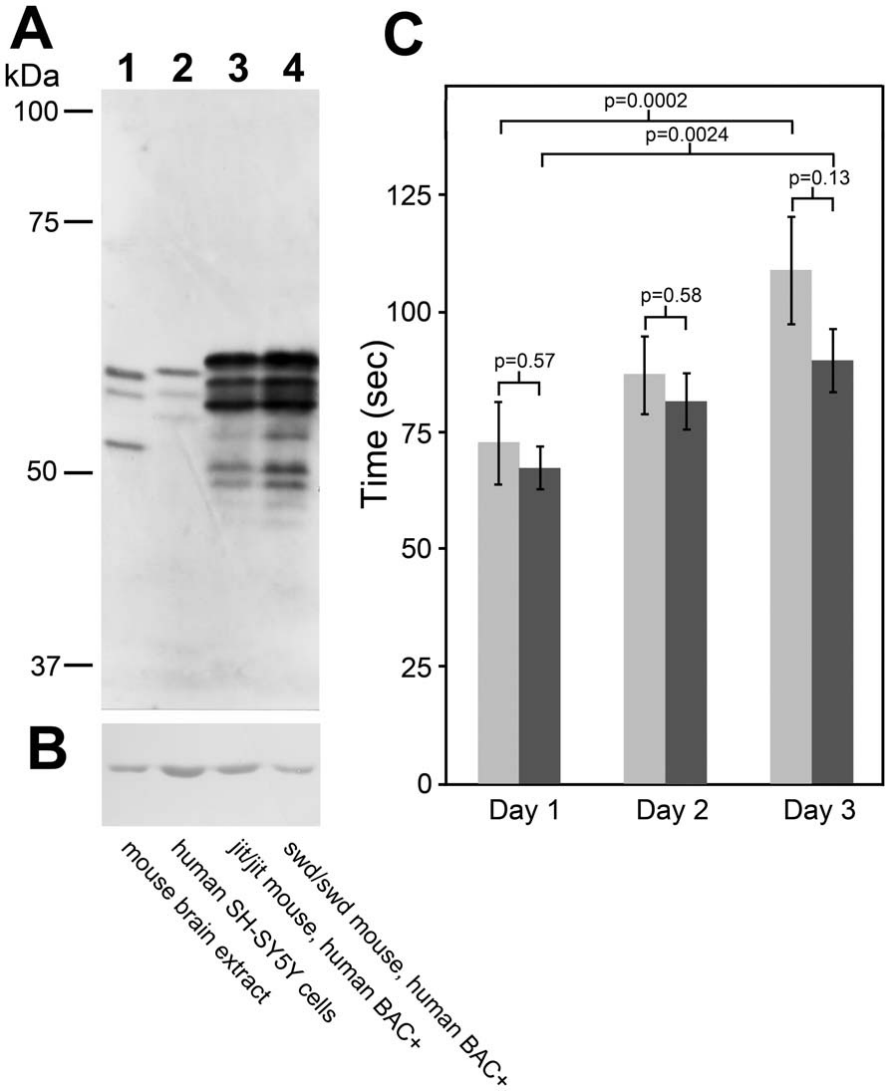
Caytaxin protein expression and functional rescue in transgenic human *ATCAY* BAC^+^ mice. (**A**) Western blot with 30 µg protein loaded, probed with anti-Caytaxin mAb 8F4 and developed using ECL. Lane 1, wild type mouse brain; lane 2, human SH-SY5Y cell lysate; lane 3, total brain protein from *ji/ji ATCAY* BAC^+^; and lane 4, *swd*/*swd ATCAY* BAC^+^. (**B**) Shows a segment of the immunoblot shown in (A) that was probed with an antibody specific for beta-actin. (**C**) Average time on a rotating rod per day, over 3 days. Wild type control +/*swd* BAC- mice (light grey bars, n = 7), and transgenic rescue *swd*/*swd* BAC^+^ mice (dark grey bars, n = 10). P-values between the control and the rescued group performance and between Day 1 and Day 3 are indicated above the columns.

To examine this hypothesis, we performed genetic complementation tests to assess the ability of human *ATCAY* DNA to rescue the severe ataxic phenotype of *Atcay* mouse mutants. Transgenic mice were generated that express a Bacterial Artificial Chromosome (BAC) containing *Homo sapiens* chromosome 19 clone CTB-171N13, which includes the complete coding region for the human *ATCAY* gene and at least 20 kb of upstream sequence; this is expected to be sufficient to direct tissue-specific expression irrespective of insertion location. These mice were bred with the two severely ataxic lines *sidewinder* and *jittery* to obtain two different transgenic BAC lines homozygous for the respective mutant alleles and positive for two independent BAC integrations (*swd*/*swd* BAC^+^ & *ji/ji* BAC^+^). These mice did not express mouse Caytaxin, but over-expressed human Caytaxin (Figure 5A, lanes 3 & 4) with no ectopic expression in non-neuronal organs, such as heart, lungs, liver, kidney, or spleen (data not shown). In lanes 3 & 4 of Figure 5, minor protein bands are visible and we attribute these additional bands to non-specific protein degradation that is caused by the overexpression of the Caytaxin protein. The pattern of the Caytaxin isoforms expressed from the BAC was consistent with the human Caytaxin protein pattern as detected in human neuroblastoma cells (Fig. 5A, lane 2). However, the size of the Caytaxin protein from BAC transgenic mice was slightly larger than endogenous Caytaxin from human neuronal cell lines. This was caused by a duplication of exon 10 in human *ATCAY* DNA, likely during the bacterial cloning phase of the BAC (data not shown). This duplication of exon 10 results in an insertion of 9 amino acids at the C-terminus of Caytaxin, but does not affect the reading frame. As a preliminary mapping of the epitopes that are recognized by the anti-Caytaxin mAbs places them further upstream, this duplication should have no impact on the staining intensity in the Western blot shown in Fig. 5A.

Initial observations of movement and motor coordination in *swd*/*swd* BAC^+^ and *ji*/*ji* BAC^+^ mice suggested gross phenotype rescue and functional complementation. Mice containing the human *ATCAY* BAC on an *Atcay* mutant background exhibited no visible signs of ataxia or dystonia (Movie S3), no difficulties accessing food or water, successfully bred and reared offspring, and lived beyond 1.5 years without gait disturbances (data not shown). To confirm and quantify this phenotypic rescue, 3–5 month-old mixed-gender *swd*/*swd* BAC^+^ and +/*swd* wild type controls were tested in a rotarod performance test. The experimental setup was designed to examine both motor coordination and learning functions, two major phenotypic aspects displayed by human individuals with Cayman ataxia [2]. We recorded the length of time that individual mice stayed on an accelerating rotating rod, with each mouse tested three times per day over a total of three days. Homozygous mutant mice that do not express the human *ATCAY* BAC are completely unable to coordinate grip, walk, or balance (Movies S1). Additionally, without special housing conditions and specific accommodations to allow access to food and water, these mice usually die after 3 weeks [4]. As *swd*/*swd* mutant mice are unable to stay on a static rod for the required 30 seconds prior to the start of the testing period, it was not possible to test their performance in this experimental paradigm.

Results obtained from the rotarod tests confirmed our earlier phenotype observations. We detected no significant difference (p>0.05 in each trial) in either motor coordination or learning and memory in *swd*/*swd* BAC^+^ mice when compared to wild type controls (Fig. 5C). *swd*/*swd* BAC^+^ mice were not only capable of maintaining their balance, but they were also able to adjust their balance when the rod began to accelerate. Their total time on the rotating rod was comparable to wild type controls (Fig. 5C). In addition, rotarod performance for both wild type and BAC-rescued mice significantly increased between the first and third day of testing, reflecting their ability to learn from previous testing experiences.

## Discussion

Individuals suffering from Cayman ataxia are homozygous for two mutations in the *ATCAY* gene and display marked motor and cognitive defects [2]. However it is unknown how these two mutations that are always found in all affected individuals affect protein expression and cause ataxia. Genetic data gathered from this rare disease have been instrumental in discovering Caytaxin, a neuronal protein important for normal nervous system function [1], [2], [5], [24]. While work has been published that characterizes Caytaxin protein localization and its interactions with other proteins [7], [8], [9], the biological function of Caytaxin remains largely unknown.

We report the use of monoclonal antibodies specific for Caytaxin to investigate Caytaxin protein expression in mutant *Atcay* mouse models. Our results demonstrate that mutations in the *Atcay* gene cause decreases in Caytaxin expression, and that the severity of ataxia is correlated with Caytaxin protein expression. Additionally, our data from transgenic *Atcay* mutant mice over-expressing the human *ATCAY* gene suggest that the high sequence homology between human and mouse Caytaxin also reflects a conservation of protein function.

While two of our monoclonal antibodies are specific for Caytaxin (Fig. 1), their utility is limited to detecting Caytaxin protein on Western blots. *In vivo* staining experiments indicated that the third anti-Caytaxin monoclonal antibody 4E3 might also cross-react with unidentified protein(s) and we therefore did not use this antibody for further analyses (data not shown). Previous reports utilized anti-Caytaxin polyclonal antibodies that were raised against small regions of the Caytaxin protein or antibodies against affinity-tagged Caytaxin. These reports found Caytaxin protein to be expressed as one or two bands on Western blots [5], [11], [12], [13], [15], [16], [25], with the doublet hypothesized to be a result of protein post-translational modifications (PTM) or RNA splicing [15]. Our analysis reveals that in one avian and several mammalian species, Caytaxin is actually expressed as three distinct isoforms of approximately 58, 55, and 50 kDa, which are all derived from a single transcript. Due to the small difference in size between these protein bands, complete resolution of all three Caytaxin isoforms requires large SDS-PAGE gels (∼10″ long separating gel), high acrylamide concentrations (10–12%), and a longer run-time at a constant current (15–30 mA). It may be for these reasons that the multiple isoforms of the Caytaxin protein have not been identified previously.

Our initial investigation into the origin of these three Caytaxin isoforms revealed that Caytaxin is one of few proteins consisting of multiple isoforms that are generated by different methionine translational start sites (Fig. 3C & D). This phenomenon is more often found in yeast proteins as well as in transcription factors and oncogenes [26] such as the cell-regulation gene c-Myc, which encodes three c-Myc protein isoforms originating from non-AUG and AUG codons [27], [28]. We identified two independent AUG start codons directly responsible for the production of the two larger Caytaxin isoforms. Although we were unable to identify the source of the smallest Caytaxin isoform, the results of our *in vitro* translation assays strongly suggest that it is not the result of mRNA splicing or processing. Further examination into the origin of the third variable isoform ruled out a third ATG start site, non-specific post-translational protease degradation, and specific carboxy-terminal cleavage of a larger Caytaxin protein species (data not shown).

Common non-methionine start sites downstream of the second conserved methionine were also examined, but yielded inconclusive results (data not shown). Interestingly, the size of the smallest isoform varies between species, pointing to the possibility of proteolytic processing. Since the expression of the smallest isoform appears highly dependent on N-terminal Caytaxin sequence and the translation of the largest protein forms (Fig. 3), we hypothesize that translation of this isoform depends on protein folding and/or the accessibility of specific protease cleavage sites during this folding process. The significance of three distinct proteins produced from a single transcript remains unknown. It will be of interest to elucidate whether the different Caytaxin protein isoforms differ in molecular function, protein-protein interactions, and/or subcellular localization. Once the nature of the third Caytaxin isoform has been revealed, it should be possible to introduce each isoform separately into transgenic mice and to analyze its individual ability to rescue Caytaxin loss-of function conditions.

Assessment into whether expression of total Caytaxin protein is modulated during mouse postnatal development revealed consistent expression of individual isoforms, but variation in total protein levels from postnatal day 1 throughout adulthood. The peaks observed in Caytaxin protein expression throughout development closely follow developmental milestones and major mouse life events such as eye opening at Day 13, weaning at Day 21, and sexual maturity at 5 weeks (Fig. 4, lanes 4–8) [29] – all of which induce changes in neuronal gene expression [30], [31], [32]. Reports from Hayakawa *et al.* also found disparity between mRNA levels and Caytaxin protein expression upon examination of *Atcay* mRNA levels throughout development [16]; this indicates that our observed fluctuations in protein levels may be due to protein processing or degradation. Further investigation towards understanding the underlying pathways, which regulate the Caytaxin gene and its protein product, will be instrumental for pinpointing the role of this protein in nervous system development.

Previous studies examining Caytaxin protein expression and phenotype in animal models have been limited to a single report in the dystonic rat line [5]. Our group sought to expand the availability of representative models through the characterization of the three *Atcay* mutant mouse lines. While the level of severity of the ataxic phenotype has been described in these mice [1], [4], there has been no published molecular characterization of Caytaxin that linked protein expression with the observed phenotype. We report that all three mice represent appropriate models for studying Caytaxin protein due to their unique mutations and corresponding Caytaxin expression. The *jittery* and *sidewinder* mice, which display a severe ataxic/dystonic phenotype [4], do not produce any Caytaxin protein. These mice usually die at an age of 3 to 4 weeks. If they are provided with softened, easily accessible food, they can survive considerably longer (data not shown). In contrast, the low levels of normal *Atcay* RNA expressed by the mildly ataxic/dystonic *hesitant* mice are sufficient for producing small amounts of normal Caytaxin protein (Fig. 1D). As described earlier [4], the phenotype of *hesitant* mice is affected by the genetic background and varies from barely noticeable ataxia to strikingly severe dystonia. Yet it is always considerably milder than in *jittery* or *sidewinder* mice. This was also observed in the complementation test when the *hesitant* allele was crossed onto a *jittery* background reducing its genetic contribution by half [4].

This correlation between Caytaxin expression and phenotype severity is also observed in our humanized transgenic *sidewinder* and *jittery* mice, which overexpress human Caytaxin and no longer display early lethality or the severe ataxic phenotype. Based on our Western blot data and quantitative PCR analysis, these transgenic mice harbor ∼12 copies of the *ATCAY*-containing BAC and over-express human Caytaxin protein by approximately 11-fold compared to wild type controls (data not shown). This over-expression does not appear to result in any leaky expression in non-neuronal tissues (data not shown) or aberrant gain-of-function phenotype (Movie S3). However, whether Caytaxin expressed from a single copy of the *ATCAY* transgene is sufficient to rescue the ataxic phenotype, similar to *sidewinder* and *jittery* heterozygotes, remains unclear. It should be noted that while our transgenic mice express *ATCAY* containing a duplication of exon 10 (Fig. 5A), the insertion occurs downstream of the BCH domain and does not affect the amino acid composition of this region (data not shown). However, it cannot be excluded that a small detrimental effect of the exon 10 duplication might be masked or compensated for by the overexpression of the protein. Conservation of the BCH domain appears to maintain Caytaxin function as these mice shown no indication of Cayman ataxia disease symptoms such as decreased motor coordination and learning functions [2] (Fig. 5C).

Data obtained from the three *Atcay* mutant mouse models indicate that Caytaxin is critical for normal nervous system function, but a relatively small Caytaxin expression level is sufficient for normal functionality. Heterozygous sidewinder mice display decreased levels of Caytaxin compared to homozygote wild type littermates with no obvious effect on phenotype (Fig. 1A). The data from the *hesitant* mice suggest that even a 10-fold reduction in Caytaxin protein does not alter motor control, coordination, or survival post-weaning. This suggests that a minimum level of Caytaxin protein is required and sufficient for normal nervous system function. Additionally, analysis of the transgenic BAC mice indicates a conservation of Caytaxin function between human and mice. Phylogenetic and evolution mapping of BNIP proteins and the BCH domain of Caytaxin suggest that this protein may play a similar role within the nervous systems of many related vertebrates and potentially also invertebrates [10]. Thus, the conserved function of Caytaxin may extend beyond *Homo sapiens* and *Mus musculus* to include other Caytaxin orthologs as potential animal models.

This report introduces new tools and methods to examine Caytaxin expression and function in complex animal systems. These approaches revealed that Caytaxin is actually expressed as multiple isoforms. It remains to be elucidated whether these multiple Caytaxin isoforms also impart multiple protein functionalities. Our report also represents the first study characterizing Caytaxin expression in mutant *Atcay* mice and demonstrates that the severity of their ataxia is correlated with Caytaxin protein levels. The conservation of function between human and mouse Caytaxin confirms the validity of mouse models for use in understanding the etiology of Cayman Ataxia as a human neurological condition.

## Materials and Methods

### Mice

The *sidewinder* (*Atcay^swd^*) line was generated from mice harboring the *ji^swd^* allele, which arose at the Jackson Laboratory in 1999, on a C57BL/6J background, and were maintained in our lab on that background. The *hesitant* mice (C3H-*Atcay^ji-hes^*/J, stock number 001904) were obtained from the Jackson Laboratory and bred in-house. *Jittery* (B6.C(Cg)-*Atcay^ji^*/BurJ, stock number 008140) were produced by our lab and donated to the Jackson Laboratory in 2007. The line was created by crossing inbred *jittery*/*grizzled* mice (JIGR/DnJ, stock number 000572) obtained from the Jackson Laboratory with C57BL/6J mice, pups from which were selected for the *ji* allele. *ji*-only heterozygotes were backcrossed with C57BL/6J over 15 generations. F_2_ crosses were performed as previously described [4], and mutant lines were maintained by heterozygote crosses. The *ATCAY*-containing BAC construct consisted of *Homo sapiens* chromosome 19 clone CTB-171N13 (GenBank: AC011488.7) in vector pBeloBACII (Research Genetics, Huntsville, Alabama). The University of Michigan Transgenic Animal Model Core purified the BAC’s and generated transgenic mice according to their protocols (http://www.med.umich.edu/tamc/tgoutline.html). The University of Michigan Unit for Laboratory Animals provided husbandry services for all mouse lines and the University of Michigan Committee on Use and Care of Animals approved all mouse experiments.

### Copy Number Assay

A BAC copy number standard was generated based on the protocol provided by The University of Michigan Transgenic Animal Model Core (http://www.med.umich.edu/tamc/spike.html). Genomic DNA from BAC transgenic mice was spiked into 5 µg of wt/wt *sidewinder* genomic DNA at concentrations equivalent to 1, 5, 10, 50, 100, 500, and 1000 copies. Concentrations were determined by calculating the mass of transgene DNA per microgram of wt/wt *sidewinder* genomic DNA with the assumption that the haploid content of the murine genome is 3×10^9^ bp. Quantitative PCR was performed using GusB as a reference gene to determine dCt’s. A standard curve was generated by plotting average dCt vs Log_2_(Copy Number) for each 1, 5, 10, 50, 100, 500, and 1000 copy number reference.

### Cell Culture

SH-SY5Y cells were obtained from Dr. Stephen K. Fisher at the University of Michigan and maintained in complete growth medium (1∶1 mixture of DMEM and F12 medium, with 10% FBS) in 5% CO2 at 37°C. Cells were sub-cultured using the recommended ATCC protocol, with some modifications. Briefly, non-adherent cells were recovered from the media, adherent cells were rinsed with HBSS (Fisher Scientific, Cat. No. SH3058801) and incubated in HBSS containing 0.25% trypsin (Life Technologies, Cat. No 15090-046) and 0.53 mM EDTA until detached. Adherent cells were combined with recovered non-adherent cells and sub-cultivated at a ratio of 1∶50. Growth medium was renewed every 4–7 days.

Cell pellets harvested for Western blots using: AtT20 (mouse corticotroph pituitary tumor) at 2×10^6^ cells; PC12 (rat pheochromocytoma) at 1×10^6^ cells; C6 (rat astrocytoma) at 2×10^6^ cells; 132-1N1 (human glioma) at 10×10^6^ cells; A172 (human glioma) at 1×10^6^ cells; N2A (mouse neuroblastoma) at 1×10^6^ cells; SH-SY5Y (human neuroblastoma) at 1×10^6^ cells.

Cell line sources: SH-SY5Y (ATCC®: CRL-2266™) AtT20 (ATCC®: CRL-1795™), PC12 (ATCC®: CRL-1721™), C6 (ATCC®: CCL-107™), 132-1N1 (Sigma-Aldrich® Cat. No. 86030402), A172 (ATCC®: CRL-1620™), N2A (ATCC®: CCL-131™).

### Genotyping

Mouse tail biopsies were performed between post-natal day 14–16 and genomic DNA was extracted using the PUREGENE® DNA Purification Kit according to the manufacturer’s protocol (Gentra, Cat. No. 158222 or 158267). Genomic PCR’s for all strains were performed by amplifying a target region from 25 ng of genomic DNA with the following buffer conditions: Expand Long Template System for *sidewinder* (Roche, Cat. No. 11681834001); PCR Buffer Set (Roche, Cat. No. 11699121001) for *jittery*; PCR Buffer set (Invitrogen, MgCl_2_ Cat. No. Y02016 and 10× PCR buffer Cat. No. Y02028) for *hesitant*. Following the *sidewinder* genomic PCR, a subsequent restriction digest with enzyme MslI (New England Biolabs, Cat. No. R0571S) was performed at 37°C for 9 hours, followed by heat inactivation of the enzyme for 20 minutes at 65°C. Agarose gel electrophoresis was performed using a 2% (*hesitant, jittery*) or 3% (*sidewinder*) sodium-borate agarose gels. *Sidewinder* and *jittery* transgenic BAC lines were genotyped for mutant alleles (described above) and the presence of the BAC transgene using primers for both exons 2 and 6 of *ATCAY*.

*sidewinder* primers: 5′-atcataggggagcaagagcatc-3′, 5′-gatggactgacagcacactgg-3′

*jittery* primers: 5′-ccctgaccacaccctcaat-3′, 5′-tttggtccagggagatgttg-3′, 5′-ctggctgtcctggaactcac-3′

*hesitant* primers: 5′-cctccctgcacagacacaatag-3′, 5′-gggatgttagggtttaccacca-3′, 5′-tacaacagaattccagggtcca-3′

*ATCAY* exon 2 primers: 5′-catggggtagacgattgtcatt-3′, 5′-acagagaagactcgcacacagg-3′

*ATCAY* exon 6 primers: 5′-aggactctgacgttgccgat-3′, 5′-tagggccacaatgcaatcct-3′

### Antibodies

A construct to create a GST-human Caytaxin fusion protein was generated by excising a 1.3 kb fragment from human *ATCAY* cDNA clone 4153341 (Life Technologies) with SalI and EcoRI restriction enzymes, and cloning it into the pGEX vector (GE Healthcare Life Sciences). The GST-human Caytaxin fusion protein was created by expressing this vector in bacteria and purifying it by preparative SDS-PAGE. The purified human Caytaxin fusion protein was used as an immunogen in mice and spleen cells from an immunized mouse were fused with myeloma cells for generating anti-Caytaxin secreting hybridoma cell lines according to the University of Michigan Hybridoma Core Facility protocols (http://www.med.umich.edu/mdrtc/cores/hybridomaCore/services.htm). Hybridoma supernatants were screened by Western blot analysis on total wild type mouse brain protein extracts and selected for specific reactivity compared with positive and negative control antibodies (Fig.S1). Three hybridoma lines 1E2, 4E3, and 8F4 were selected, sub-cloned and used for ascites production by The University of Michigan Hybridoma Core Facility (http://www.med.umich.edu/mdrtc/cores/hybridomaCore/index.html). Except where indicated, all anti-Caytaxin monoclonal antibodies were used at a dilution of 1∶500. The anti-actin antibody was used for Western blotting according to the manufacturer’s protocol (monoclonal mouse anti-beta-actin from Sigma, Cat. No. A1978). Mouse-anti-beta tubulin E7 antibody was donated by Dr. Diane Fingar at the University of Michigan and used at 1∶1000 or 1∶2000. Peroxidase-conjugated AffiniPure Goat anti-mouse IgG antibodies were used per manufacturer’s protocol (Jackson ImmunoResearch Laboratories, Inc. Cat. No. 115-035-062).

### Tissue Lysate Preparation

All protein extractions were performed on ice with pre-chilled reagents and materials. Tissues were homogenized in a glass dounce homogenizer in 1–2 mL of homogenization buffer (50 mM Tris, 500 mM NaCl, 1 mM EDTA, 0.5% NP40, 1× Protease Inhibitor Cocktail (Thermo Scientific), 1× PMSF, 0.1% SDS or 2% SDS). Homogenization was performed for 1–2 minutes (approximately 50 strokes). Homogenate was centrifuged at 4°C in a pre-cooled microcentrifuge at 15,000×g for 10–15 minutes to clear large debris. Supernatant was used immediately or flash-frozen with liquid nitrogen and stored at −80°C. Protein concentrations were measured using a commercial Bradford Assay (Bio-Rad, Cat. No. 500-0006).

### SDS-PAGE

All Western blot SDS-PAGE gels were large (separating gel 25 mL volume, ∼10 inches in length, 1 mm thick), and prepared to 10% polyacrylamide except where indicated. Samples were heated at 100°C for 5–10 minutes in 2X Laemmli buffer with β-mercaptoethanol. Gels were run in 1X Tris-Glycine-SDS running buffer at a constant current of 15–30 milliamps and electrophoretically transferred at a constant current of 100 milliamps overnight at room temperature in 0.5X Tris-Glycine buffer onto a nitrocellulose membrane (Pall Life Sciences, BioTrace™ NT, Cat. No. 66485). Ponceau S staining of nitrocellulose membranes was performed according to the manufacturer’s protocol (USB, Cat. No. 32819, prepared as directed) to confirm transfer of proteins onto the membrane.

### Immunoblotting

*ECL:* Membranes were incubated in blocking buffer (5% wt/vol non-fat dry milk powder in TBST) for at least 1 hour. Primary and secondary antibodies were diluted in blocking buffer and subsequently incubated with intermediate washes for 1 hour at room temperature. All washes were performed 3 times, 10 minutes each, in TBST. ECL (GE/Amersham, Cat. No. RPN2106 or Fisher Scientific/Pierce, Cat. No. PI32106), autoradiography blue film (Fisher Scientific, Cat. No. NC9469626 or AF5700). *DAB:* Performed as described by Hortsch *et al* [33]. Briefly, membranes were incubated in blocking buffer (1×PBS, 10% FCS, 0.05% TX100). Primary and secondary antibodies were diluted in blocking buffer to 1∶1000 and 1∶2000 respectively. Membranes were washed in blocking buffer after primary antibody incubation and in wash buffer (1×PBS and 0.05% TX100) after secondary antibody incubation followed by 1×PBS. Membranes were briefly rinsed with 50 mM Tris-HCl before development with DAB (50 mM Tris-HCl pH 7.5, 0.05% diaminobenzidine, 30% H_2_O_2_). Reactions were stopped by rinsing membrane in dH_2_O.

### Phosphorylation and Ubiquitination Analyses

Ubiquitination was examined using an anti-ubiquitin antibody (Ubiquitin polyclonal antibody, Boston Biochem® Cat. No. A-100) against both mouse Caytaxin from whole brain lysates and human Caytaxin from SH-SY5Y cell line lysates. Ubiquitin protein was included as a positive control (Mammalian Ubiquitin, Boston Biochem® Cat. No. U-100). Samples were run on a large, 22% urea gel, and detected with ECL. For determining the Caytaxin protein phosphorylation status, Caytaxin protein was immunoprecipitated from wild type mouse brain lysates using anti-Caytaxin monoclonal antibody 4E3 and Protein G beads. Samples were treated with lambda phosphatase (Lambda protein phosphatase, New England Biolabs, Cat. No. P0753S) at 200U/reaction, and then separated on a 10% SDS-PAGE gel followed by Western blot analysis using ECL. For a positive control, anti-S6 Kinase antibodies were used on insulin-stimulated HEK293 cell lysates.

### cDNA Constructs and Sequence Alignment

Human *ATCAY* cDNA and mouse *Atcay* cDNA were obtained from Open Biosystems (Cat. No. MHS1010-7429547, Clone ID 4153341 and Cat. No. MMM1013-9200338, Clone ID 6491141, respectively). The RefSeq number for the mouse *Atcay* cDNA construct is NM_178662.3, GenBank: BC048903.1 and for the human *ATCAY* construct is NM_033064.4, GenBank: BC026217.1.

### Site-directed Mutagenesis

Mutagenic primer pairs were designed using Agilent Technologies web-based QuikChange® Primer Design Program (http://www.genomics.agilent.com).

Met1Thr Sense 5′-ctctttccagctctcacgggaaccacagaagc-3′

Anti 5′-gcttctgtggttcccgtgagagctggaaagag-3′

Sense 5′-gaagctacactaaggacggaaaatgtggacgtgaggg-3′

Anti 5′-ccctcacgtccacattttccgtccttagtgtagcttc-3′

Sense 5′-gctctcacgggaaccacagaagctacactaaggacggaaaatg-3′

Anti 5′-cattttccgtccttagtgtagcttctgtggttcccgtgagagc-3′

Reaction setup and PCR cycling parameters were followed according to the manufacturer’s protocol with 25 ng template DNA, 125 ng primers, and an 8 minute PCR extension (QuikChange II Site Directed Mutagenesis Kit, Agilent Technologies, Cat. No. 200519). Mutagenesis reactions were transfected into XL1-Blue Competent Cells (Agilent Technologies, Cat. No. 200249) and plated on nutrient agar with 100 ug/mL ampicillin. Positive clones were identified via miniprep (Qiagen, according to manufacturer’s protocol) followed by sequencing using primer T7 (5′-taatacgactcactataggg-3′).

### In Vitro Translation

Coupled transcription/translation reactions were set up according to manufacturer’s protocol using the TnT® T7/SP6 Coupled Reticulocyte Lysate System (Promega, Cat. No. L5020). 7 µL of a 25 µL reaction was loaded on 10% SDS-PAGE gels. Additional reagents included EasyTag™ L-[^35^S]-Methionine, 500µCi (18.5 MBq) (PerkinElmer, NEG709A500UC) and RNasin® Ribonuclease Inhibitor (Promega, Cat. No. N2111). Gels were fixed overnight in 50% MeOH and 10% acetic acid, and then treated with EN^3^HANCE™ Autoradiography Enhancer according to the manufacturer’s protocol (PerkinElmer, Cat. No. 6NE9701). Gels were dried on a slab gel dryer for 2 hours at 80°C and exposed to autoradiography blue film (Fisher Scientific, Cat. No. NC9469626 or AF5700) for 1–7 days at room temperature. *Atcay/ATCAY* cDNA inserts were correctly oriented from the SP6 promoter. Reactions with T7 RNA polymerase served as negative controls.

### Rotarod

Animals were required to perch on a stationary rod for approximately 30–60 seconds to accustom themselves to the environment. The animals that were comfortable staying on the rod were allowed to run with a constant speed of 5 rpm for approximately 60 seconds. Animals that fell after three trials with constant low speed rotation (2 rpm) were removed from the experiment. When test animals were able to stay on the rod for approximately 30 seconds, they were allowed to return to their home cage. Animals were trained again after at least 2 hours of rest or the following day. During the third training session, the rod was allowed to accelerate until the animal fell. Those mice that passed initial testing requirements underwent testing with 3 trials per day over 3 days, for a total of 9 runs. 7 wt/swd BAC^-^ and 10 swd/swd wtBAC^+^ mice were tested, ages 3–5 months with mixed gender, and time on the rod for each test animal was recorded.

### Graphs and Statistics

All graphs were created using Microsoft Excel and p-values were calculated using Student’s t-test. Error bars were calculated based on standard deviation from the mean (SEM) of the corresponding group. Protein quantification was performed after scanning sub-saturationally-exposed films by analysis with ImageJ (http://rsbweb.nih.gov/ij/) and relative ratios were calculated based on density.

### In silico Analyses

Sequence alignments, Caytaxin protein translation start site predictions, and Caytaxin molecular weight calculations were analyzed using sequences obtained from the NCBI database: *Homo sapiens* ataxia, cerebellar, Cayman type (*ATCAY*) mRNA: NM_033064.4 and Caytaxin protein: NP_149053.1; *Mus musculus* ataxia, cerebellar, Cayman type homolog (human) (*Atcay*) mRNA: NM_178662.3 and Caytaxin protein: NP_848777.1.

Potential translation start codons were analyzed using the NetStart 1.0 Prediction Server (http://www.cbs.dtu.dk/services/NetStart/). Met1 and Met10 were identified as the top two candidates with scores of 0.806 and 0.744 respectively [34]. The ATGpr prediction program (http://atgpr.dbcls.jp/) also identified Met1 and Met10 as the top two candidates with 0.70 and 0.31 reliability [35]. Mouse and human Caytaxin protein molecular weight was calculated using the SIB Swiss Institute of Bioinformatics “Compute pI/Mw” online tool *(* http://web.expasy.org/compute_pi/) [17]. Sequence similarity calculations were performed by alignment using NCBI’s BLAST®.

## Supporting Information

Figure S1 **Anti-Caytaxin monoclonal antibody screen.** Western blots with wild type mouse whole brain protein extracts were used to screen hybridoma supernatants for anti-Caytaxin antibody activity. Lanes 2–7 were developed with DAB. Lane 1, total wild type mouse brain protein as detected by Ponceau S staining; lane 2, pattern of polyclonal mouse serum from mouse sacrificed for the fusion protocol; lanes 3, 4 and 5, patterns from hybridoma wells 1E2, 4E3, and 8F4 respectively; lanes 6 and 7, hybridoma supernatants 4E9 and 8G8 were later determined not to contain any anti-Caytaxin activity, but rather reacted with unknown proteins of different molecular weights.
(TIF)

Figure S2 **Caytaxin protein expression in major brain regions.** Western blot with 30 µg of total protein extracts from different brain regions of a 25-month-old wild type mouse, developed with ECL. Lane 1, hippocampal area; lane 2, frontal lobe; lane 3, cerebellum; and lane 4, residual brain matter. The blot was probed with anti-Caytaxin mAb 8F4 (upper panel) and an anti-actin antibody (lower panel).
(TIF)

Movie S1 **Movie of** ***Atcay swd*** **mutant mouse phenotype.** This video shows a *sidewinder* heterozygote (*swd*/wt), displaying no ataxia, and a homozygote mutant (*swd*/*swd*) littermate with severe ataxia and limited mobility and motor coordination.
(WMV)

Movie S2 **Movie of** ***Atcay hes*** **mutant mouse phenotype.** This video shows a homozygous *hesitant* mutant animal (*hes*/*hes*) with mild ataxia and dystonia, an exaggerated stepping due to apparent muscle tension, slight defect in motor coordination, and subtle defects in balance.
(WMV)

Movie S3 **Movie of rescued** ***Atcay*** **mutant mouse phenotype.** This video shows a homozygous *sidewinder* mutant mouse expressing human *ATCAY-*BAC (*swd*/*swd* BAC^+^) with no ataxia phenotype and a homozygous *sidewinder* mutant littermate, which does not have the human *ATCAY*-BAC (*swd*/*swd* BAC-) and displays a *sidewinder* mutant phenotype seen in Movie S1.
(WMV)
