# Supplementary figures and images for: Expression of Caytaxin Protein in Cayman Ataxia Mouse Models Correlates with Phenotype Severity
Source: PLoS One. 2012 Nov 30;7(11):e50570. doi: 10.1371/journal.pone.0050570 (PMC3511541; doi:10.1371/journal.pone.0050570)

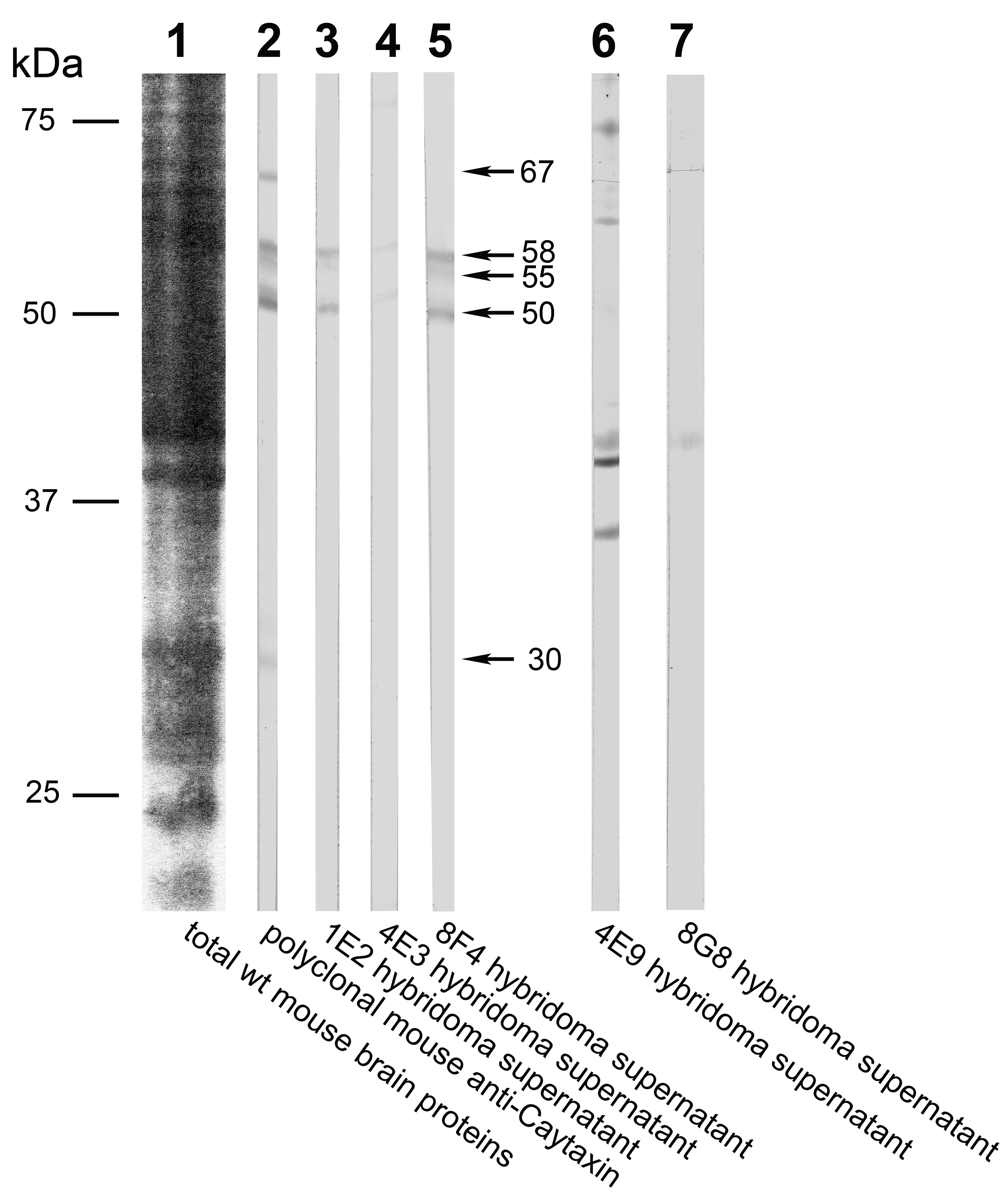

Supplement: Figure S1 — Anti-Caytaxin monoclonal antibody screen. Western blots with wild type mouse whole brain protein extracts were used to screen hybridoma supernatants for anti-Caytaxin antibody activity. Lanes 2–7 were developed with DAB. Lane 1, total wild type mouse brain protein as detected by Ponceau S staining; lane 2, pattern of polyclonal mouse serum from mouse sacrificed for the fusion protocol; lanes 3, 4 and 5, patterns from hybridoma wells 1E2, 4E3, and 8F4 respectively; lanes 6 and 7, hybridoma supernatants 4E9 and 8G8 were later determined not to contain any anti-Caytaxin activity, but rather reacted with unknown proteins of different molecular weights. (TIF) [file pone.0050570.s001.tif]

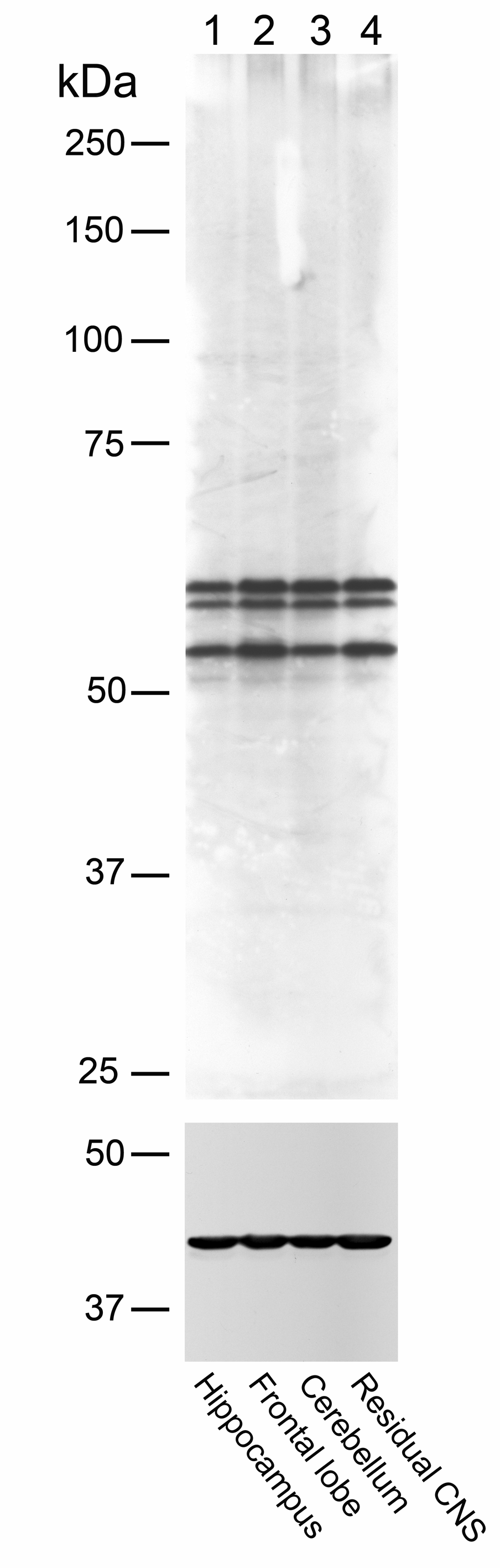

Supplement: Figure S2 — Caytaxin protein expression in major brain regions. Western blot with 30 µg of total protein extracts from different brain regions of a 25-month-old wild type mouse, developed with ECL. Lane 1, hippocampal area; lane 2, frontal lobe; lane 3, cerebellum; and lane 4, residual brain matter. The blot was probed with anti-Caytaxin mAb 8F4 (upper panel) and an anti-actin antibody (lower panel). (TIF) [file pone.0050570.s002.tif]
